# High prevalence of germline *STK11 *mutations in Hungarian Peutz-Jeghers Syndrome patients

**DOI:** 10.1186/1471-2350-11-169

**Published:** 2010-11-30

**Authors:** Janos Papp, Marietta Eva Kovacs, Szilvia Solyom, Miklos Kasler, Anne-Lise Børresen-Dale, Edith Olah

**Affiliations:** 1 Department of Molecular Genetics, National Institute of Oncology, Budapest, Hungary; 2 Laboratory of Cancer Genetics, Department of Clinical Genetics and Biocenter Oulu, University of Oulu, Oulu University Hospital, Oulu, Finland; 3 Department of Head and Neck Surgery, National Institute of Oncology, Budapest, Hungary; 4 Department of Genetics, Institute for Cancer Research, Oslo University Hospital Radiumhospitalet, Oslo, Norway; 5 Institute for Clinical Medicine, Faculty of Medicine, Univeristy of Oslo, Norway

## Abstract

**Background:**

Peutz-Jeghers syndrome (PJS) is a rare autosomal dominantly inherited disease characterized by gastrointestinal hamartomatous polyposis and mucocutaneous pigmentation. The genetic predisposition for PJS has been shown to be associated with germline mutations in the *STK11*/*LKB1 *tumor suppressor gene. The aim of the present study was to characterize Hungarian PJS patients with respect to germline mutation in *STK11*/*LKB1 *and their association to disease phenotype.

**Methods:**

Mutation screening of 21 patients from 13 PJS families were performed using direct DNA sequencing and multiplex ligation-dependent probe amplification (MLPA). Comparative semi-quantitative sequencing was applied to investigate the mRNA-level effects of nonsense and splice-affecting mutations.

**Results:**

Thirteen different pathogenic mutations in *STK11*, including a high frequency of large genomic deletions (38%, 5/13), were identified in the 13 unrelated families studied. One of these deletions also affects two neighboring genes (*SBNO2 *and *GPX4*), located upstream of *STK11*, with a possible modifier effect. The majority of the point mutations (88%, 7/8) can be considered novel. Quantification of the *STK11 *transcript at the mRNA-level revealed that the expression of alleles carrying a nonsense or frameshift mutation was reduced to 30-70% of that of the wild type allele. Mutations affecting splice-sites around exon 2 displayed an mRNA processing pattern indicative of co-regulated splicing of exons 2 and 3.

**Conclusions:**

A combination of sensitive techniques may assure a high (100%) *STK11 *mutation detection frequency in PJS families. Characterization of mutations at mRNA level may give a deeper insight into the molecular consequences of the pathogenic mutations than predictions made solely at the genomic level.

## Background

Peutz-Jeghers Syndrome (PJS) is a rare autosomal-dominant hereditary condition with incomplete penetrance, characterized by hamartomatous polyps of the gastrointestinal tract and pigmented lesions of the buccal mucosa, perioral region and other sites [1,2]. PJS patients have an increased risk of cancer of multiple locations, predominantly the colon, small intestine, stomach, esophagus, pancreas, breast, ovary and uterine cervix [3-6].

This condition is primarily associated with germline mutations in the serine/threonine kinase 11 (*STK11*/*LKB1*) gene, localized on the chromosomal segment 19p13.3 [7,8]. The gene spans 23 kb, consists of nine coding exons and a final noncoding exon [9,10]. The protein it codes for plays a role in cellular energy metabolism, cell polarization, p53-dependent apoptosis, VEGF regulation and Wnt signal transduction [11-14].

The prevalence of germline pathogenic *STK11 *point mutations in PJS cases has been reported with very different frequencies, ranging from about 90% to only 10% [9,10,15], depending on both patient selection criteria and the screening methods used. The low point mutation rates reported in some studies raised the possibility of the existence of another PJS susceptibility gene, a notion investigated by genetic linkage analyses in PJS families [16,17]. An alternative hypothesis, offering an explanation for the absence of *STK11 *mutations in PJS families in some studies, is the existence of alterations not detectable by the conventional mutation screening methods used. Multiplex Ligation-dependent Probe Amplification (MLPA) proved to be a powerful, robust and easy-to-perform approach to scan for large genomic deletions, and this type of mutation was indeed shown to have a significant contribution to the *STK11 *mutation pattern [18-20]. Taken together, *STK11 *aberrations seem to date to account for almost all familial PJS cases, bringing the existence of genetic heterogeneity into question.

We report here the clinicopathological manifestation and results of a comprehensive mutation analysis of the *STK11 *gene in 13 unrelated PJS families. We describe a number of novel mutations in PJS patients comprising the largest number of patients from the Central-Eastern European region reported so far.

## Methods

### Patients and samples

Individuals in this study were referred for genetic counseling and testing to the Department of Molecular Genetics at the National Institute of Oncology, Budapest, Hungary between 1995 and 2008. All investigations have been carried out in compliance with internationally recognized guidelines. Study protocols have been approved by the Institutional Ethical Board. Written informed consent was obtained from each patient. Included in this study were 21 patients from 13 Peutz-Jeghers families.

### Mutation analysis

DNA was extracted from blood samples of all consenting subjects using the classic phenol-chloroform method. The entire coding region and splice junctions of the *STK11 *gene were amplified by PCR (primer sequences are available upon request). Systematic mutation screening was performed using direct bidirectional sequencing applying an ABI 3130 Genetic Analyzer (Applied Biosystems). The presence of all mutations was confirmed using a different blood sample.

Additionally, the coding region of *STK11 *was screened for genomic aberrations using the MLPA Kit P101 (MRC-Holland), according to the manufacturer's recommendations, and as described previously [21]. For the determination of the exact lengths of deletions having both breakpoints within the gene, we applied a combination of XL-PCR and sequencing. Since the various deletions detected all required individual approaches to determine the exact length of the deletion, a detailed description is outlined for each of them in the legends of the figures (Additional files 1 and 2: Figures S1 and S2).

To assess the approximate length of deletions extending over the 5' gene boundaries, the sequence copy number at multiple sites upstream of *STK11 *was determined. Gene dosage assays were performed in triplicates using the Power SYBR Green PCR Master Mix on an ABI Prism 7900 HT Sequence Detection System (Applied Biosystems). The conditions for thermal cycling were 50°C for 2 min and 95°C for 10 min, followed by 40 cycles of 95°C for 15 sec and 60°C for 1 min. The regions used for copy number testing were 16.4 kb, 31.6 kb, 99.4 kb, 110.5 kb, and 120.1 kb upstream of the *STK11 *gene, all selected from non-repetitive regions. Primer sequences and localization data are given in Additional file 3: Table S1. The ratio changes between these 5' and known two-copy regions were calculated by the 2^-ΔΔCt ^method [22] using mutation-negative samples for calibrations after ensuring that amplification efficiencies of the control and target amplicons were comparable.

The mutation nomenclature used here complies with the recommendations of den Dunnen and Antonarakis [23,24], sequence variations are named in relation to the ATG codon in cDNA reference sequence NM_000455.4, and predicted changes at the protein level are given according to the protein reference sequence NP_000446.1, as detailed on the website of the Human Genome Variation Society [25].

### Expression analysis

We performed RNA extraction from blood leukocytes of mutation carriers using the RNAqueous Kit, and carried out cDNA synthesis starting from 400 ng of total RNA using the High-Capacity cDNA Reverse Transcription Kit according to the manufacturer's instructions (Applied Biosystems). In order to assess the effects of the mutations at the mRNA level, we sequenced the cDNA of carrier individuals and estimated the expression levels of the mutant alleles by comparing the area-under-the-curve ratios at heterozygous mutation positions, using the genomic DNA sample as calibrator.

### Statistical analysis

Differences between groups were calculated using a Student's t-test, with p values less than 0.05 considered significant.

## Results

### Patient characteristics

A total of 13 probands and 8 affected relatives with a history of Peutz-Jeghers syndrome (PJS) were included in this study. The disease was diagnosed at an average age of 21.9 years (within a range of 3-51 years). All but one of the probands showed the classical PJS phenotype of buccal freckling and hamartomatous polyps; one proband (HP09), diagnosed at 35 years of age, showed no typical mucocutaneous pigmentation. From the 13 probands only 8 had a family history of the disease, while the remaining 5 cases (38%) appeared to be the result of *de novo *mutations. These two groups showed no significant difference in their average age of disease onset (20.7 years for those with family history versus 22.2 years for the suggested *de novo *mutation carriers; p = 0.41). Clinical characteristics of the patients in this study are detailed in Table 1.

**Table 1:** Clinical characteristics of patients diagnosed with Peutz-Jeghers syndrome

Family	Patient	**Gender**^**a**^	**Age**^**b**^	**Localization**^**c**^	Polyp count	**PP**^**d**^	Family history
HP01	4688	F	29	SB	40-50	+	dizygotic twins
	4689	F	29	SB	60-70	+	

HP02	6107	M	33	SB	7	+	-

HP03	6202	M	24	SB	<10	+	uncle &
	6135	M	13	SB	<10	+	nephew

HP04	6213	M	22	ST;SB	>100	+	-

HP05	6456	F	22	CR	<10	+	mother &
	6457	F	8	ST	1	+	daughter

HP06	6453	F	11	ST;SB;CR	<50	+	-

HP07	6525	M	10	ST;SB;CR	>100	+	-

HP08	6782	M	27	ST	<10	+	son &
	6783	F	31	SB	<10	-	mother

HP09	6853	M	35	ST;SB;CR	>100	-	-

HP10	7081	M	3	*ND*	*ND*	+	father &
	7082	F	4	*ND*	*ND*	+	daughter

HP11	7132	F	14	SB	>10	+	siblings
	7130	F	11	SB;CR	>10	*ND*	

HP12	5829	F	26	SB	<50	+	mother &
	5116	F	11	SB	<10	+	daughter

HP13	6800	F	51	SB;CR	>10	+	mother &
	6799	M	28	SB	2	+	son

a: F = female; M = male

b: Age at diagnosis (years)

c: Polyp localization, CR = colorectum; SB = small bowel; ST = stomach

d: presence of perioral pigmentation

ND: no available data

### Germline mutations

**Figure 1:**
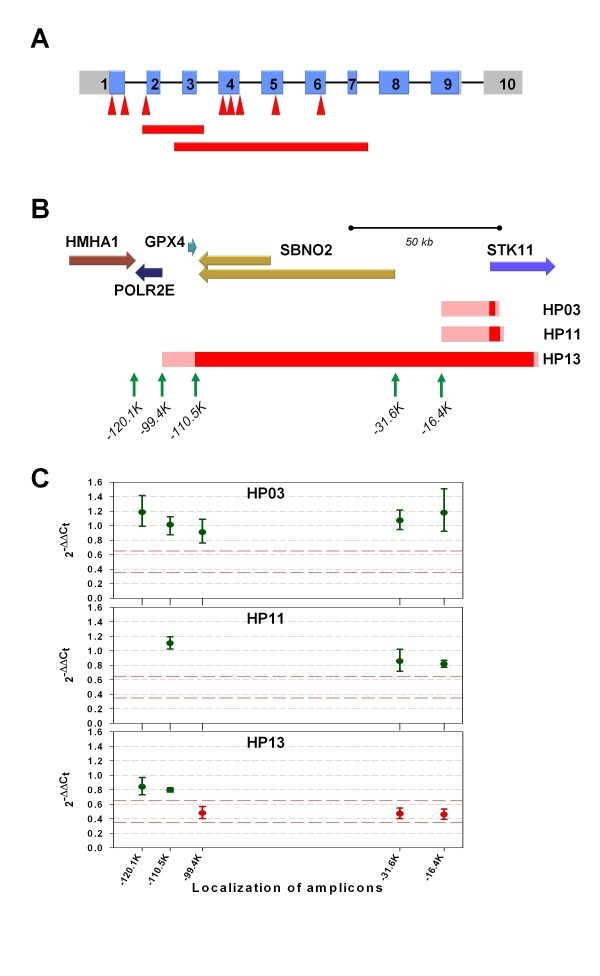
**Germline mutations in the STK11 gene**. Panel A shows the mutations within the gene: exon structure of the STK11 gene is at the top, the coding region is shown in blue. The introns are not drawn to scale. The locations of point mutations are marked by red arrowheads; the intragenic large deletions are depicted as red rectangles below the gene. The approximate localization of large deletions extending outwards STK11 is portrayed on Panel B. The known RefSeq genes of the region are shown as filled arrows. The minimal and maximal sizes of the large genomic deletions are indicated as red and pink bars, respectively. The loci where copy number analyses were done are marked by vertical green arrows, names reflecting their localization with respect to the STK11 gene. Panel C shows the data of the gene dosage experiments.

From the 13 unrelated families enrolled in this study, 13 distinct pathogenic mutations were indentified. These included three one-base deletions leading to frameshifts, three nonsense base changes, two substitutions affecting splice sites and five genomic deletions removing one to seven exons; three of this latter type of aberrations extended into the upstream genomic regions of the *STK11 *gene (Table 2), one of them also affecting two neighboring genes (*SBNO2 *and *GPX4*). A schematic representation of the mutations uncovered is presented in Figure 1, showing that all of them affect the kinase domain of the protein.

**Table 2:** Germline STK11 mutations in PJS patients

Family	Exon/Intron	Mutation name	**Mutation type**^**a**^	**Effect on cDNA or mRNA/protein level**^**b**^
HP01	ex 2-3	c.291-5484_464+384del6865	GD	exon 2-3 skipping
HP02	ex 6	c.801delC	FS	p.Ile267MetfsX20
HP03	ex 1	c.1-?_290+?del	GD	(no start; 1 allele)
HP04	ex 1	c.180C>A	NS	p.Tyr60X
HP05	ex 7	c.876C>G	NS	p.Tyr292X
HP06	ex 1	c.142A>T	NS	p.Lys48X
HP07	ex 4	c.550delC	FS	p.Leu184SerfsX103
HP08	in 1	c.291-2A>T	SS	*exon 2-3 skipping*
HP09	ex 4	c.540delG	FS	p.Asn181ThrfsX107
HP10	ex 3-7	c.375-106_921-264del3504insA	GD	*exon 2-7 skipping*
HP11	ex 1-3	c.1-?_464+?del	GD	(no start; 1 allele)
HP12	in 4	c.597+1G>A	SS	exon 4 skipping
HP13	ex 1-7	c.1-?_920+?del	GD	(no start; 1 allele)

a: GD = genomic deletion; FS = frameshift mutation; NS = nonsense mutation; SS = splice-site mutation

b: predicted effects are shown in brackets; mutation effect on mRNA-level disagreeing with previous predictions are shown in italics

**Figure 2:**
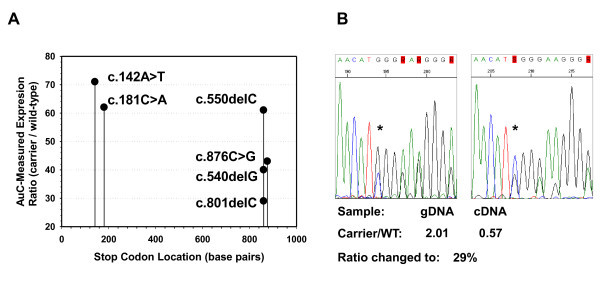
**Demonstration of the decreased expression of alleles carrying nonsense and frameshift mutations**. Panel A shows the results of semiquantitative sequencing of six STK11 mutations. The relative expression of the mutant allele (based on area-under-the-curve measurements, AuC) is shown in relation to the nucleotide position of the newly acquired stop codon. An example of AuC measurement is shown on Panel B. The genomic DNA and cDNA sequencing electrophoretograms of a c.801delC mutation carrier sample (HP02) is presented, with the AuC base ratio as well as the ratio change (cDNA compared to gDNA) given below for the nucleotide position marked by an asterisk.

Reduced mRNA expression level of the mutant allele was observed for all cases carrying point mutations causing premature stop codons (small deletions and nonsense changes). This reduction of allelic expression showed a broad range (30-70% mutant allele level in cDNA, as compared to the corresponding genomic DNA) and was more prominent for the new acquired stop codons closer to the 3' end of the gene. Interestingly, the three mutations (c.540delG, c.550delC and c.801delC) giving rise to the same premature stop codon (in exon 6) also showed a comparatively wide spectrum of decrease in the expression of the mutant allele (30-60%) (Figure 2).

Bioinformatics analysis predicted the two splice-site affecting mutations (c.291-2A>T and c.597 + 1G>A) to abrogate normal splicing (a decrease in splice site score from 9.4 to 1.6 for the 3' site in intron 1 and from 6.5 to 4.2 for the 5' site in intron 4 as calculated by the online tool hosted by the Zhang Lab [26]). Indeed, cDNA analysis revealed that the intron 4 nucleotide substitution results in skipping of exon 4, while the c.291-2A>T mutation resulted in a loss of both exons 2 and 3, demonstrated at the cDNA level (data not shown).

In the cases with genomic deletions where both 5' and 3' breakpoints were situated within the gene, junction determination revealed different sequence features at the boundaries. The deletion encompassing exons 2-3 in HP01 most probably occurred by homologous recombination between two Alu elements in the same strand (AluJr in intron 1/AluY in intron 3). The breakpoints of the other deletion (removing exons 3-7 in HP10) are not localized to Alu elements or any other repetitive sequences, but revealed the addition of one nucleotide at the junction site, (close to but not within a microhomology). See Additional files 1, 2, 4 and 5: Figures S1, S2, S3 and S4) for more details on the determination of genomic deletion breakpoints.

**Figure 3:**
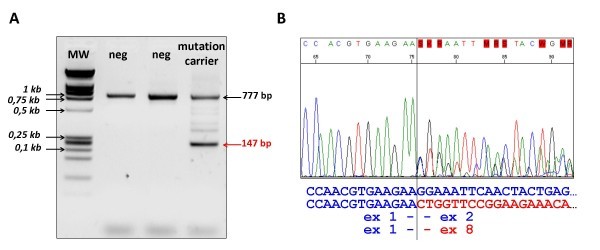
**PCR analysis and sequencing results of the proband of family HP10 presenting with a large genomic deletion encompassing exons 3-7**. Panel A shows the results of the PCR amplification of the cDNA sample of the mutation carrier along with samples from two negative controls (designated as 'neg'), using a sense primer in exon 1 and an antisense primer in exon 8. The extra band in the mutation carrier sample (marked by a red arrow) indicates the presence of a variant mRNA species resulting from fusion of exon 1 to exon 8. Faint bands on the gel are most likely the results of heteroduplex molecules. MW: molecular weight marker; neg: mutation negative samples. The sequencing result illustrating the skipping of exons 2-7 is shown on Panel B with the sequences of the normal as well as the mutant allele given below the sequenogram.

Interestingly, cDNA analysis of these cases with deletions showed a splicing pattern of exon 2-3 skipping on cDNA of HP01, confirming the alteration seen at the genomic (DNA) level. For the carrier of the genomic deletion of exons 3-7 we found that exons 2 to 7 were skipped from the cDNA, inconsistent with the initial prediction of exon 3-7 cDNA loss (Figure 3).

## Discussion

In most previous studies germline *STK11 *mutations were found in 50-90% of PJS patients [20,27]. In this study we identified deleterious sequence alterations in all 13 PJS families studied, including point mutations (8/13, 62%) as well as large genomic deletions removing from one to several exons of the gene (5/13, 38%). This high fraction of large genomic aberrations identified in Hungarian patients is in agreement with the proportions seen in several other populations [18,19,28,29]. Only one of the point mutations uncovered in our cohort (c.180C>A; p.Tyr60X) can be found in the publicly available version of The Human Genome Mutation Database [30,31], all other point mutations can be considered novel. The novel or recurrent status of the large genomic deletions cannot be safely assessed, since the exact breakpoint sequences of these are usually left unidentified in the literature except for in two very recent publications [32,33]. According to these cited studies, Alu elements show a striking overrepresentation in the genomic region where *STK11 *resides, and play a major role in its instability, although deletion mechanisms not involving repetitive elements are also common [32,33]. Our data are in line with these recent results, since out of the two deletions for which we were able to determine the breakpoints, one junction involved two Alu elements with a 26 bp perfect homology, suggesting the mechanism of homologous recombination. The other deletion showed the insertion of one nucleotide at the junction site, which is a common feature of NHEJ repair. Unfortunately, we could not determine the exact breakpoints of the other deletions due to the difficulties in amplifying the GC- and repeat-rich regions involved.

The extent of large genomic aberrations involving other tumor suppressor genes is also only rarely determined, although these deletions may affect other neighboring genes having a potential phenotype-modifying effect [33-35], sometimes even raising the possibility of novel mutation mechanisms [36]. In our PJS families, out of the 5 large deletions one extended into the coding region of other genes situated upstream of *STK11*, as determined by copy-number assays at specific genomic loci. In this case (HP13), the deletion affected the 5' genes *SBNO2 *and *GPX4*, but did not reach the next upstream gene *POLR2E*. The gene *SBNO2*, a component of the IL-10-mediated anti-inflammatory pathway [37] has currently no known association with gastrointestinal diseases [33], but it was affected together with *STK11 *in several cell lines investigated [38,39]. Also, interleukin and JAK-STAT signaling, in which SBNO2 plays a role [37], is widely implicated in gastrointestinal cancers. Interestingly, a polymorphism at the 3' UTR of the glutathione peroxidase *GPX4 *was recently reported as a risk-modifier for colorectal cancer [40], the allele associated with lower *GPX4 *expression being linked to lower cancer risk. Taking these results into account, it is possible that the complete inactivation of one allele of this gene by a deletion may have a similar protective effect on the disease phenotype. Indeed, the age at disease onset was higher for both affected members of the HP13 family (28 and 51 years of age) as compared to the average age of onset of all other PJS patients (19.1 years), but no other specific feature was detected. Our results are comparable with those reported by Le Meur and co-workers [41], who characterized a large genomic deletion in one family completely removing one copy of the *STK11 *gene and several other genes upstream of it (including *GPX4*). In that study, two relatives of the proband were diagnosed with PJS at the relatively late age of 43 years, suggesting a potential modifier role for *GPX4 *in PJS.

In order to assess the potential of aberrant *STK11 *mRNA species to produce truncated proteins in blood leukocytes, we determined the allelic ratio of mutation carrier transcripts by semi-quantitative sequencing and found that their amount ranged from ~30-70% compared to their wild-type counterpart. Despite the semi-quantitative nature of this method, our results are similar to those obtained by other groups examining mutated alleles of several different genes [42,43]. The reduction of the level of premature stop codon-containing transcripts is partially attributable to the mechanism of nonsense-mediated mRNA-decay, an evolutionarily conserved mechanism distinguishing normal from premature termination codons on the basis of their location with respect to exon-exon junctions, and targeting the latter for degradation [44]. Moreover, the fact that three different frameshift mutations giving rise to the same early stop codon presented with highly dissimilar levels of the mutant allele either reflects on the inter-individual variability in NMD efficiency [45-47], or it may imply that mechanisms other than nonsense-mediated mRNA decay play a role in determining the fate of these transcripts.

Another mechanism preventing the emergence of truncated Stk11 protein forms is that most known *STK11 *mutations, and all mutations mentioned in the present study, destroy the kinase domain of the protein, which in turn prevents the binding of Hsp90 and Cdc37, two proteins indispensable for stabilizing Stk11 [48,49].

Aside from the decreased stability of the mutant transcripts, mRNA-level characterization of the carriers' samples revealed another interesting feature. The effect of the c.291-2A>T mutation destroying the consensus sequence of the intron 1 splice acceptor site was predicted to be the loss of exon 2, but both exons 2 and 3 were skipped in the mutant mRNA; likewise, the genomic deletion of exons 3-7 caused exon 2-7 skipping. This pattern of closely coupled splicing of two adjacent exons might be associated with the fact that the second intron of *STK11 *is processed by the minor U12-dependent spliceosome [50]. Moreover, the linked skipping of exon 2 and 3 seems to be an evolutionarily conserved phenomenon, since this isoform is the most prominent alternatively spliced *STK11 *mRNA species listed in The Alternative Splicing and Transcript Diversity Database for both men and mice [51,52]. Its protein product has been shown to be catalytically inactive and to reside exclusively in the nucleus, in contrast to the diverse cellular localization of full-length Stk11 [48].

## Conclusions

In summary, the high detection rate of mutations in our study underlies the importance of using a combination of techniques, preferably direct sequencing and MLPA, for *STK11 *germline mutation screening in PJS patients. Analysis of mRNA also seems to be crucial to appropriately assess the consequences of mutations which may not be straightforward from bioinformatics predictions only. Our results support the idea that elucidating the role of the *GPX4 *gene and potentially also the *SBNO2 *as modifiers in PJS or in PJS-associated tumors would be of high interest.

## Competing interests

The authors declare that they have no competing interests.

## Authors' contributions

JP carried out most of the molecular genetics studies and drafted the manuscript, participated in study conception and design, data acquisition and interpretation. MEK carried out the characterization of two genomic aberrations, including sequencing the deletion breakpoints; SS uncovered one of the germline mutations, participated in the expression studies and helped in critical revision of the manuscript. MK participated in the analysis of clinical data. EO participated in the conception, design and coordination of the study; recruited patients and samples for the study; participated in the collection, management, analysis and interpretation of the data. ALBD provided useful discussion and criticism. All authors read and approved the final version of the manuscript.

## Pre-publication history

The pre-publication history for this paper can be accessed here:

http://www.biomedcentral.com/1471-2350/11/169/prepub

## Supplementary Material

Additional file 1 **XL-PCR analysis of two samples carrying the genomic deletion of exons 2-3 of the STK11 gene**. The results of the XL-PCR amplification of the genomic deletion is shown. Click here for file

Additional file 2 **Determination of the length of the genomic deletion removing exons 3-7 of the STK11 gene**. The results of the MLPA and PCR analyses of the genomic deletion is shown. Click here for file

Additional file 3 **Primers used for dosage assays**. Primer sequences and localization information is given for all amplicons used for dosage assays of chromosome regions upstream of the STK11 gene. Click here for file

Additional file 4 **Breakpoint sequence of the genomic deletion removing exons 2-3 of the STK11 gene**. The genomic deletion breakpoint is shown on a sequencing chromatogram with additional information on the repetitive elements involved in the deletion. Click here for file

Additional file 5 **Breakpoint sequence of the genomic deletion removing exons 3-7 of the STK11 gene**. The genomic deletion breakpoint is shown on a sequencing chromatogram with additional information on the sequence elements involved in the deletion. Click here for file
