# Supplementary material for: High prevalence of germline STK11 mutations in Hungarian Peutz-Jeghers Syndrome patients
Source: BMC Med Genet. 2010 Nov 30;11:169. doi: 10.1186/1471-2350-11-169 (PMC3012662; doi:10.1186/1471-2350-11-169)
Supplement: Additional file 1 — XL-PCR analysis of two samples carrying the genomic deletion of exons 2-3 of the STK11 gene. The results of the XL-PCR amplification of the genomic deletion is shown. [file 1471-2350-11-169-S1.PPT]

## Slide 1
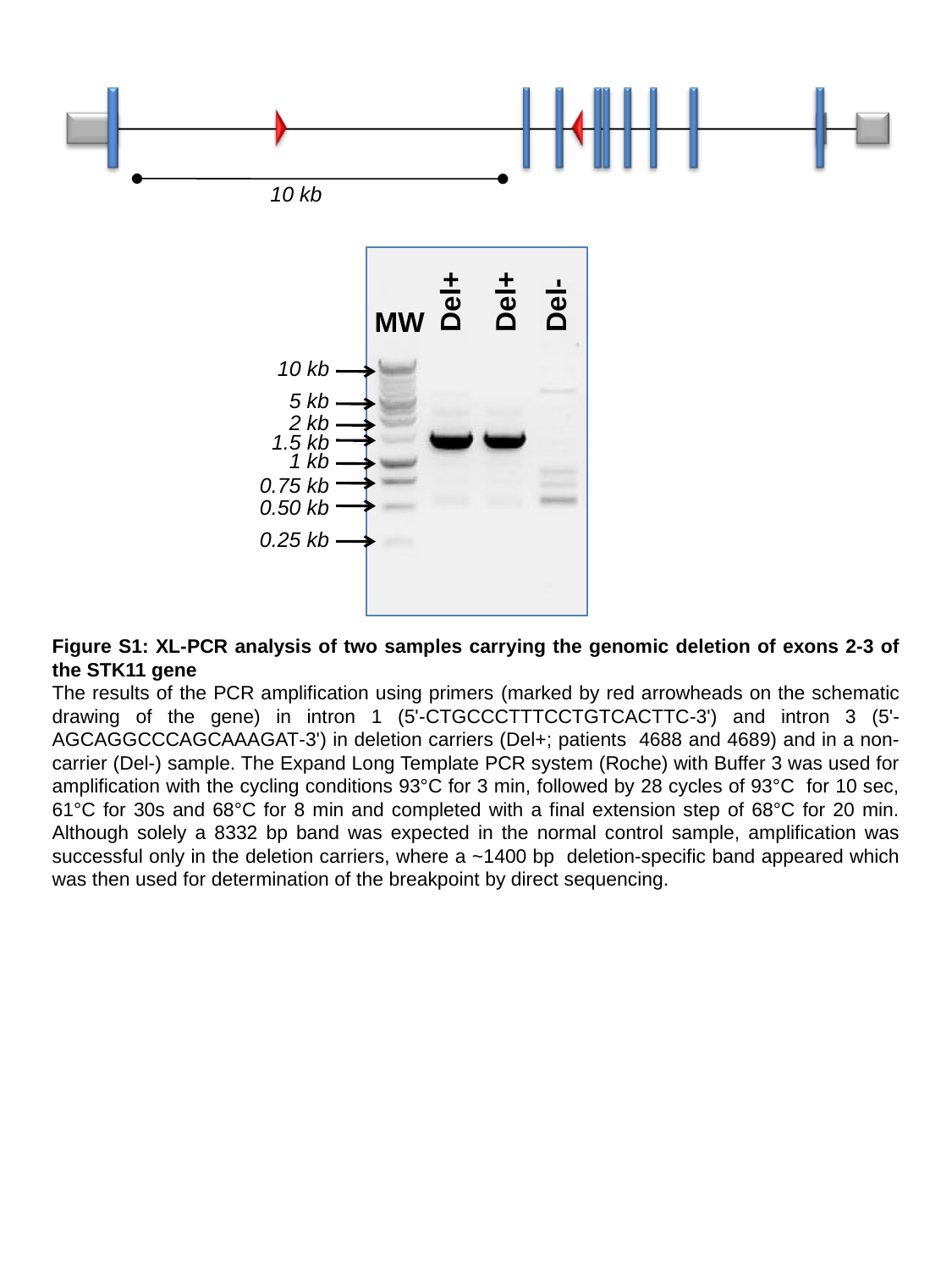

10 kb
Del+
Del+
Del-
MW
10 kb
5 kb
2 kb
1.5 kb
1 kb
0.75 kb
0.50 kb
0.25 kb
Figure S1: XL-PCR analysis of two samples carrying the genomic deletion of exons 2-3 of the STK11 gene
The results of the PCR amplification using primers (marked by red arrowheads on the schematic drawing of the gene) in intron 1 (5'-CTGCCCTTTCCTGTCACTTC-3') and intron 3 (5'-AGCAGGCCCAGCAAAGAT-3') in deletion carriers (Del+; patients 4688 and 4689) and in a non-carrier (Del-) sample. The Expand Long Template PCR system (Roche) with Buffer 3 was used for amplification with the cycling conditions 93°C for 3 min, followed by 28 cycles of 93°C for 10 sec, 61°C for 30s and 68°C for 8 min and completed with a final extension step of 68°C for 20 min. Although solely a 8332 bp band was expected in the normal control sample, amplification was successful only in the deletion carriers, where a ~1400 bp deletion-specific band appeared which was then used for determination of the breakpoint by direct sequencing.
