# Supplementary material for: High prevalence of germline STK11 mutations in Hungarian Peutz-Jeghers Syndrome patients
Source: BMC Med Genet. 2010 Nov 30;11:169. doi: 10.1186/1471-2350-11-169 (PMC3012662; doi:10.1186/1471-2350-11-169)
Supplement: Additional file 2 — Determination of the length of the genomic deletion removing exons 3-7 of the STK11 gene. The results of the MLPA and PCR analyses of the genomic deletion is shown. [file 1471-2350-11-169-S2.PPT]

## Slide 1
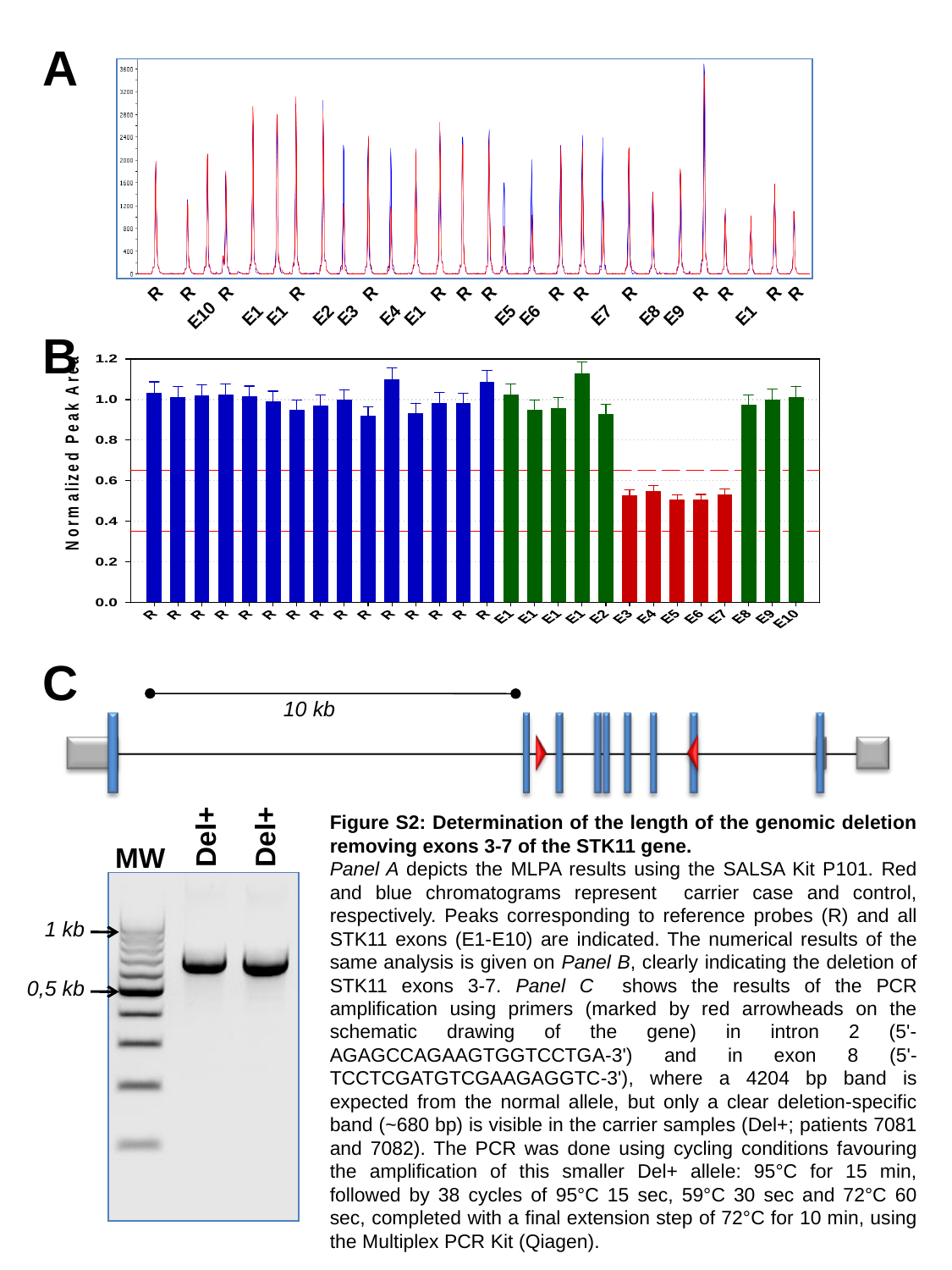

A
R
R
R
R
R
R
R
R
R
R
R
R
R
R
R
E10
E1
E1
E2
E3
E4
E1
E5
E6
E7
E8
E9
E1
B
C
10 kb
Figure S2: Determination of the length of the genomic deletion removing exons 3-7 of the STK11 gene.
Panel A depicts the MLPA results using the SALSA Kit P101. Red and blue chromatograms represent carrier case and control, respectively. Peaks corresponding to reference probes (R) and all STK11 exons (E1-E10) are indicated. The numerical results of the same analysis is given on Panel B, clearly indicating the deletion of STK11 exons 3-7. Panel C shows the results of the PCR amplification using primers (marked by red arrowheads on the schematic drawing of the gene) in intron 2 (5'- AGAGCCAGAAGTGGTCCTGA-3') and in exon 8 (5'- TCCTCGATGTCGAAGAGGTC-3'), where a 4204 bp band is expected from the normal allele, but only a clear deletion-specific band (~680 bp) is visible in the carrier samples (Del+; patients 7081 and 7082). The PCR was done using cycling conditions favouring the amplification of this smaller Del+ allele: 95°C for 15 min, followed by 38 cycles of 95°C 15 sec, 59°C 30 sec and 72°C 60 sec, completed with a final extension step of 72°C for 10 min, using the Multiplex PCR Kit (Qiagen).
Del+
Del+
MW
1 kb
0,5 kb
