# Supplementary material for: High prevalence of germline STK11 mutations in Hungarian Peutz-Jeghers Syndrome patients
Source: BMC Med Genet. 2010 Nov 30;11:169. doi: 10.1186/1471-2350-11-169 (PMC3012662; doi:10.1186/1471-2350-11-169)
Supplement: Additional file 3 — Primers used for dosage assays. Primer sequences and localization information is given for all amplicons used for dosage assays of chromosome regions upstream of the STK11 gene. [file 1471-2350-11-169-S3.DOC]

**Table S1: Primers used for dosage assays**

| **Name*** | **Localization**** | **In gene** | **Sense and antisense**  **primer sequences (5’-3’)** |
| --- | --- | --- | --- |
| **-16.4K** | chr19:1,189,443-1,189,665 | intergenic | CTCGTCGGTAAAGTGGTGGT |
|  |  |  | GACTCACCCGACTTGAGAGG |
| **-31.6K** | chr19:1,174,152-1,174,256 | SBNO2 | GTCGCAACCAGAGCCTCA |
|  |  |  | CGAAACCCGGAAGTGAGC |
| **-99.4K** | chr19:1,106,385-1,106,560 | GPX4 | CCGTCTCTCCACAGTTCCTC |
|  |  |  | GGGGCAGGTCCTTCTCTATC |
| **-110.5K** | chr19:1,095,262-1,095,461 | POLR2E | ATGATGGTCTTGCGGATTTT |
|  |  |  | GTTGCTGCATTCAGGCATT |
| **-120.1K** | chr19:1,085,657-1,085,917 | HMHA1 | AGAATCCCGAGTTGTGTCCA |
|  |  |  | ACGTTGTTGGACTGGTTGGT |

*The name of the amplicon reflects its localization with respect to the STK11 gene

** Chromosome coordinates refer to the Feb 2009 (GHCr37/hg19) human genome assembly
