# Supplementary material for: High prevalence of germline STK11 mutations in Hungarian Peutz-Jeghers Syndrome patients
Source: BMC Med Genet. 2010 Nov 30;11:169. doi: 10.1186/1471-2350-11-169 (PMC3012662; doi:10.1186/1471-2350-11-169)
Supplement: Additional file 4 — Breakpoint sequence of the genomic deletion removing exons 2-3 of the STK11 gene. The genomic deletion breakpoint is shown on a sequencing chromatogram with additional information on the repetitive elements involved in the deletion. [file 1471-2350-11-169-S4.PPT]

## Slide 1
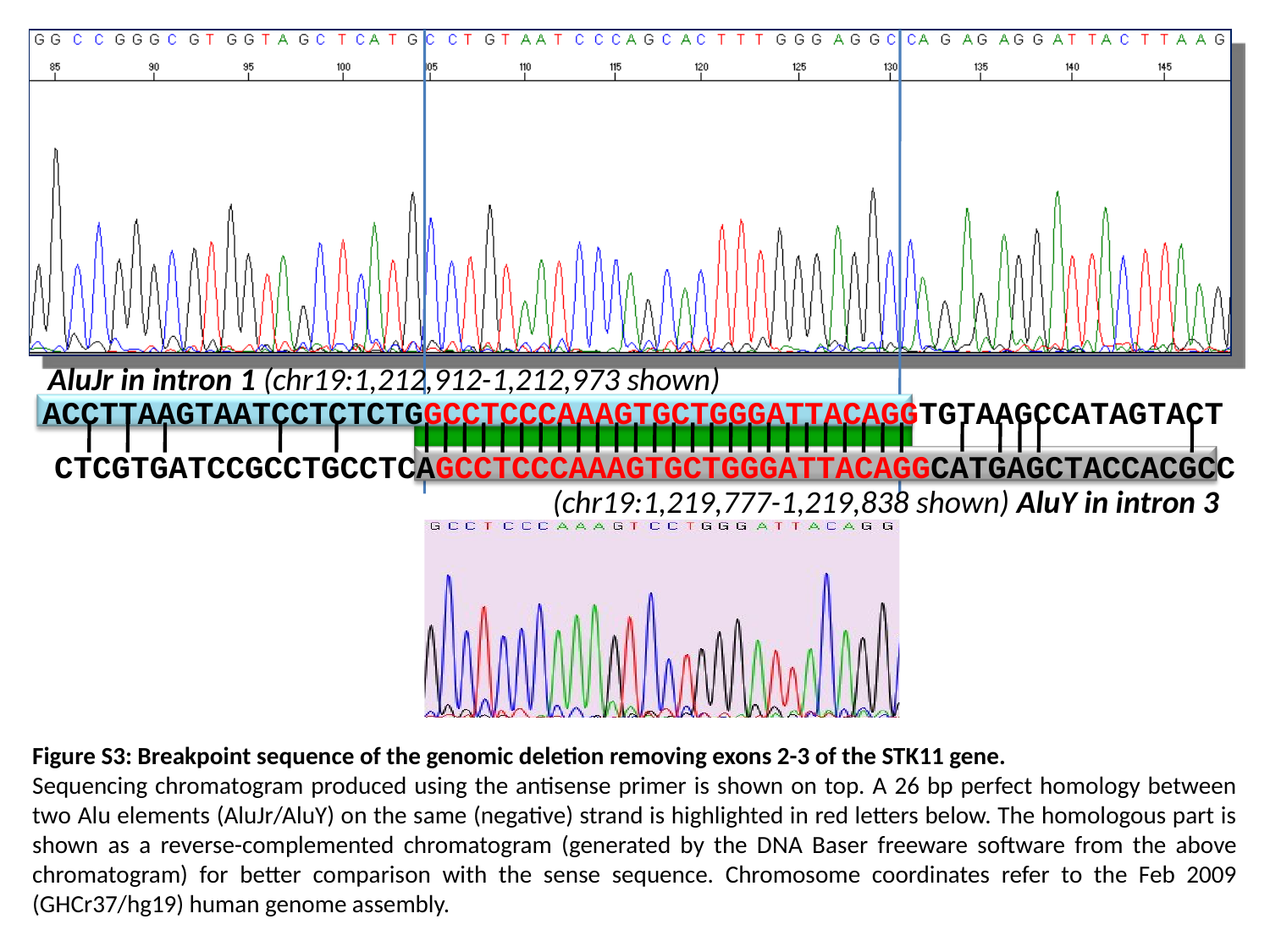

AluJr in intron 1 (chr19:1,212,912-1,212,973 shown)
ACCTTAAGTAATCCTCTCTGGCCTCCCAAAGTGCTGGGATTACAGGTGTAAGCCATAGTACT
 CTCGTGATCCGCCTGCCTCAGCCTCCCAAAGTGCTGGGATTACAGGCATGAGCTACCACGCC
(chr19:1,219,777-1,219,838 shown) AluY in intron 3
Figure S3: Breakpoint sequence of the genomic deletion removing exons 2-3 of the STK11 gene.
Sequencing chromatogram produced using the antisense primer is shown on top. A 26 bp perfect homology between two Alu elements (AluJr/AluY) on the same (negative) strand is highlighted in red letters below. The homologous part is shown as a reverse-complemented chromatogram (generated by the DNA Baser freeware software from the above chromatogram) for better comparison with the sense sequence. Chromosome coordinates refer to the Feb 2009 (GHCr37/hg19) human genome assembly.
