# Supplementary material for: High prevalence of germline STK11 mutations in Hungarian Peutz-Jeghers Syndrome patients
Source: BMC Med Genet. 2010 Nov 30;11:169. doi: 10.1186/1471-2350-11-169 (PMC3012662; doi:10.1186/1471-2350-11-169)
Supplement: Additional file 5 — Breakpoint sequence of the genomic deletion removing exons 3-7 of the STK11 gene. The genomic deletion breakpoint is shown on a sequencing chromatogram with additional information on the sequence elements involved in the deletion. [file 1471-2350-11-169-S5.PPT]

## Slide 1
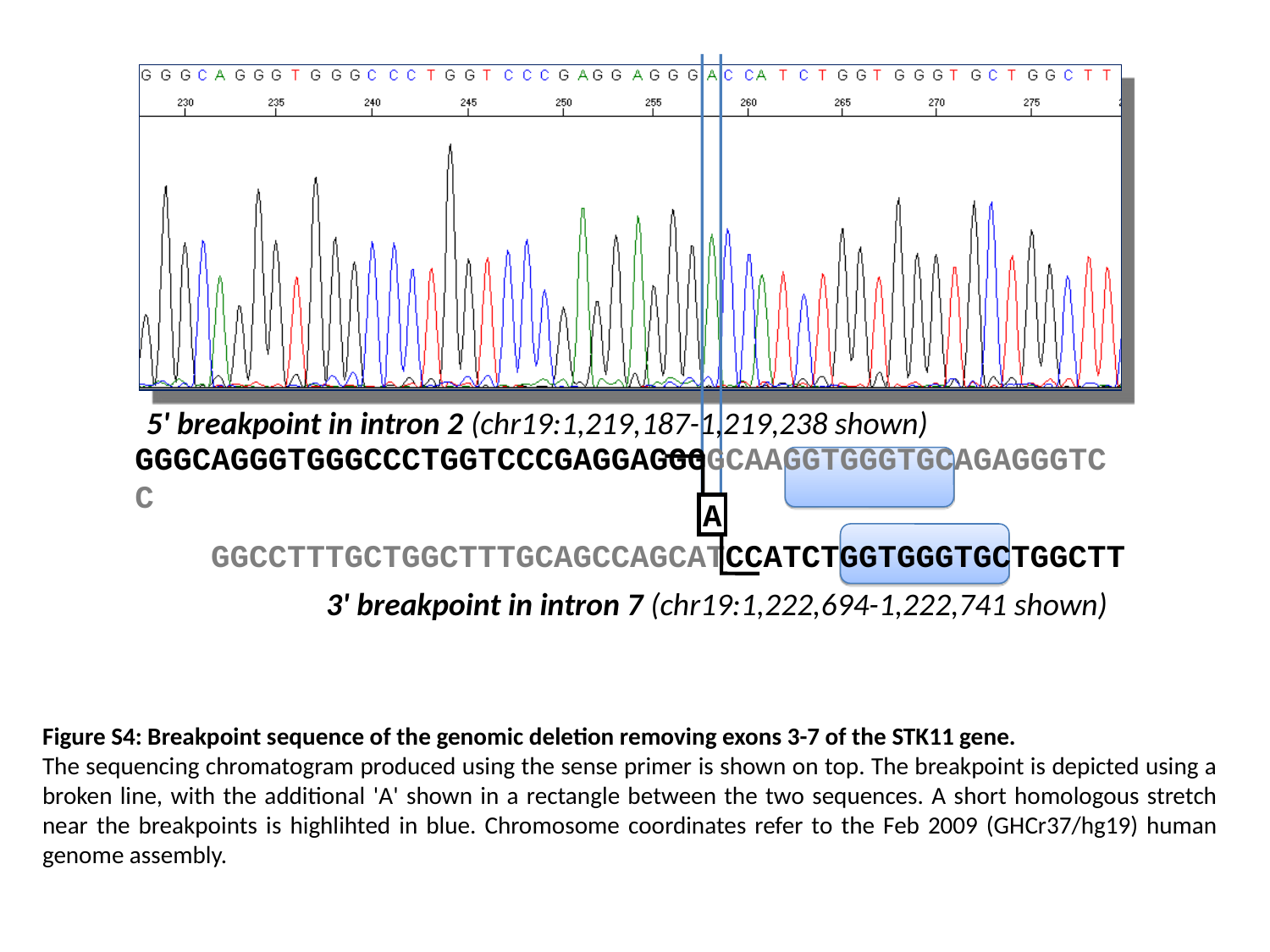

5' breakpoint in intron 2 (chr19:1,219,187-1,219,238 shown)
GGGCAGGGTGGGCCCTGGTCCCGAGGAGGGGCAAGGTGGGTGCAGAGGGTCC
A
GGCCTTTGCTGGCTTTGCAGCCAGCATCCATCTGGTGGGTGCTGGCTT
3' breakpoint in intron 7 (chr19:1,222,694-1,222,741 shown)
Figure S4: Breakpoint sequence of the genomic deletion removing exons 3-7 of the STK11 gene.
The sequencing chromatogram produced using the sense primer is shown on top. The breakpoint is depicted using a broken line, with the additional 'A' shown in a rectangle between the two sequences. A short homologous stretch near the breakpoints is highlihted in blue. Chromosome coordinates refer to the Feb 2009 (GHCr37/hg19) human genome assembly.
